# Association between Serum Perfluorooctanoic Acid (PFOA) and Thyroid Disease in the U.S. National Health and Nutrition Examination Survey

**DOI:** 10.1289/ehp.0901584

**Published:** 2010-01-20

**Authors:** David Melzer, Neil Rice, Michael H. Depledge, William E. Henley, Tamara S. Galloway

**Affiliations:** 1 Epidemiology and Public Health Group and; 2 Environment and Human Health Group, Peninsula Medical School, Exeter, United Kingdom; 3 School of Mathematics and Statistics, University of Plymouth, Plymouth, United Kingdom; 4 School of Biosciences, University of Exeter, Exeter, United Kingdom

**Keywords:** C8, human population, PFOA, PFOS, thyroid disease

## Abstract

**Background:**

Perfluorooctanoic acid (PFOA, also known as C8) and perfluorooctane sulfonate (PFOS) are stable compounds with many industrial and consumer uses. Their persistence in the environment plus toxicity in animal models has raised concern over low-level chronic exposure effects on human health.

**Objectives:**

We estimated associations between serum PFOA and PFOS concentrations and thyroid disease prevalence in representative samples of the U.S. general population.

**Methods:**

Analyses of PFOA/PFOS versus disease status in the National Health and Nutrition Examination Survey (NHANES) for 1999–2000, 2003–2004, and 2005–2006 included 3,974 adults with measured concentrations for perfluorinated chemicals. Regression models were adjusted for age, sex, race/ethnicity, education, smoking status, body mass index, and alcohol intake.

**Results:**

The NHANES-weighted prevalence of reporting any thyroid disease was 16.18% (*n* = 292) in women and 3.06% (*n* = 69) in men; prevalence of current thyroid disease with related medication was 9.89% (*n* = 163) in women and 1.88% (*n* = 46) in men. In fully adjusted logistic models, women with PFOA ≥ 5.7 ng/mL [fourth (highest) population quartile] were more likely to report current treated thyroid disease [odds ratio (OR) = 2.24; 95% confidence interval (CI), 1.38–3.65; *p* = 0.002] compared with PFOA ≤ 4.0 ng/mL (quartiles 1 and 2); we found a near significant similar trend in men (OR = 2.12; 95% CI, 0.93–4.82; *p* = 0.073). For PFOS, in men we found a similar association for those with PFOS ≥ 36.8 ng/mL (quartile 4) versus ≤ 25.5 ng/mL (quartiles 1 and 2: OR for treated disease = 2.68; 95% CI, 1.03–6.98; *p* = 0.043); in women this association was not significant.

**Conclusions:**

Higher concentrations of serum PFOA and PFOS are associated with current thyroid disease in the U.S. general adult population. More work is needed to establish the mechanisms involved and to exclude confounding and pharmacokinetic explanations.

The perfluoroalkyl acids (PFAAs) are a family of synthetic, highly stable perfluorinated compounds with a wide range of uses in industrial and consumer products, from stain- and water-resistant coatings for carpets and fabrics to fast-food contact materials, fire-resistant foams, paints, and hydraulic fluids (Organisation for Economic Co-operation and Development 2005). The carbon–fluoride bonds that characterize PFAAs and make them useful as surfactants are highly stable, and recent reports indicate the widespread persistence of certain PFAAs in the environment and in wildlife and human populations globally (Fromme et al. 2009; Giesy and Kannan 2001; Lau et al. 2007; Saito et al. 2004). Two of the PFAAs of most concern are the eight-carbon–chain perfluorooctane sulfonate (PFOS) and perfluorooctanoic acid (PFOA, also known as C8).

Most persistent organic pollutants are lipophilic and accumulate in fatty tissues, but PFOS and PFOA are both lipo- and hydrophobic, and after absorption will bind to proteins in serum rather than accumulating in lipids (Hundley et al. 2006; Jones et al. 2003). The renal clearance of PFOA and PFOS is negligible in humans, leading to reported half-lives in blood serum of 3.8 and 5.4 years for PFOA and PFOS, respectively (Olsen et al. 2007). Human biomonitoring of the general population in various countries has shown that, in addition to the near ubiquitous presence of PFOS and PFOA in blood, these may also be present in breast milk, liver, seminal fluid, and umbilical cord blood (Lau et al. 2007).

Extensive laboratory studies of the toxicology of PFOA and PFOS have reported enlargement of the liver, modulation of sex hormone homeostasis, developmental and immune system toxicity, hypolipidemia, and reduced body weight in rodent and nonhuman primate models [reviewed by Lau et al. (2004, 2007)]. Research interest has focused on the ability of these compounds to bind to nuclear receptors, including the peroxisome proliferator–activating receptor (PPARα), and to disrupt serum protein ligand binding (Luebker et al. 2002), highlighting PFOA and PFOS as potential endocrine disruptors (Jensen and Leffers 2008).

Endocrine systems that may be targets of endocrine-disrupting chemicals include the hypothalamus–pituitary–thyroid (HPT) axis (Boas et al. 2006). Thyroid hormone is essential for the normal physiologic function of nearly all mammalian tissues. Thyroid hormone status is controlled by a well-established feedback mechanism, in which thyroid-stimulating hormone (TSH) stimulates the thyroid to synthesize thyroxine (T_4_), which is then converted to the biologically active triiodothyronine (T_3_). The rate of release of TSH is regulated by the hypothalamus as well as by the circulating levels of T_3_ and T_4_. Therefore, multiple physiologic steps, including hormone biosynthesis, transport, metabolism, and action on target cells, are required for thyroid hormone homeostasis.

Numerous studies have now shown PFAAs to impair thyroid hormone homeostasis in animal studies. Depression of serum T_4_ and T_3_ in PFOS-exposed rats has been reported by several authors (Lau et al. 2003; Luebker et al. 2005; Seacat et al. 2003), without the concomitant increase in TSH that would be expected through feedback stimulation. Earlier mechanistic studies of structurally related perfluorodecanoic acid showed that it could reduce serum thyroid hormone levels apparently by reducing the responsiveness of the HPT axis and by displacing circulating thyroid hormones from their plasma protein-binding sites (Gutshall et al. 1989). Although circulating hormone levels were depressed, the activities of thyroid hormone–sensitive liver enzymes were elevated, suggesting that functional hypothyroidism was not occurring. A similar mechanism for PFOS has been hypothesized (Chang et al. 2007). A recent study of the mechanisms involved in PFOS-induced hypothyroxinemia in rats has indicated that increased conjugation of T_4_ in the liver, catalyzed by the hepatic enzyme uridine diphosphoglucuronosyl transferase (UGT1A1), and increased thyroidal conversion of T_4_ to T_3_ by type 1 deiodinase may be partly responsible for the effects (Yu et al. 2009). Taken together, these findings suggest that the effects of PFAAs on thyroid hormone physiology are multiple and complex.

Extrapolations from animal laboratory studies such as these to an estimation of the risks posed by PFOA and PFOS to thyroid function in humans are complicated by the extreme variations reported in their toxicokinetic profile between species (Johnson et al. 1979; Olsen et al. 2007). The extremely long half-lives of PFOA and PFOS in humans contrast with the relatively rapid elimination seen in animal models (e.g., serum half-life of PFOS in rats is around 100 days) (Hundley et al. 2006), drawing attention to the potential risks to human health. Disruption to thyroid hormone balance was not found in previous studies of community exposure to PFOA (Emmett et al. 2006; Olsen et al. 2003b) or PFOS (Inoue 2004). Modest associations between PFOA and thyroid hormones (negative for free T_4_ and positive for T_3_) were reported in 506 PFOA production workers across three production facilities (Olsen and Zobel 2007); there were no associations between TSH or T_4_ and PFOA, and the free hormone levels were within the normal reference range.

Given the evidence from animal studies of thyroid hormone imbalance and the varied epidemiologic results from community and occupational exposures, we aimed to explore the hypothesis that higher serum PFOA and PFOS concentrations would be associated with thyroid disease in the general adult population. The environmental chemical biomonitoring program of the U.S. Centers for Disease Control and Prevention, using samples from the U.S. National Health and Nutrition Examination Survey (NHANES), provides large-scale data on serum PFAA concentrations in population-representative samples. In this study we used these data to estimate associations between PFOA/PFOS concentrations and thyroid disease in representative samples of the U.S. general population.

## Methods

### Study population

Data were from three independent cross-sectional waves of NHANES: 1999–2000, 2003–2004, and 2005–2006. NHANES surveys assess the health and diet of the noninstitutionalized civilian population of the United States and are administered by the National Center for Health Statistics (NCHS). The study protocol for NHANES was approved by the NCHS Institutional Review Board.

### Assessment of PFOA/PFOS concentrations

Solid-phase extraction coupled to high-performance liquid chromatography/turbo ion spray ionization/tandem mass spectrometry with isotope-labeled internal standards was used for the detection of PFOA and PFOS, with a limit of detection of 0.2 ng/mL (Kuklenyik et al. 2005). The laboratory methods and comprehensive quality control system were consistent in each NHANES wave, and documentation for each wave is available (NCHS 2009; NHANES 2006, 2007).

Serum polyfluorinated chemicals (PFCs) were measured in a one-third representative random subset of persons ≥ 12 years of age in each NHANES wave. Data from individuals < 20 years of age were excluded, because questions relating to disease prevalence were asked only for adults.

### Disease outcomes

In all NHANES waves, adult respondents were asked about physician-diagnosed diseases. Associations were examined between PFOA and PFOS concentrations and thyroid disease outcomes. Individuals were asked whether they had ever been told by a doctor or health professional that they had a thyroid problem (in the 1999–2000 survey the questions related to goiter and other thyroid conditions) and whether they still had the condition. We further defined thyroid disease by considering those people who said they currently had thyroid disease and were taking any thyroid-related medication, including levothyroxine, liothyronine, “thyroid desiccated,” and “thyroid drugs unspecified” for hypothyroidism and propylthiouracil and methimazole for hyperthyroidism. No details were available on specific thyroid disease diagnosis, and the PFC samples did not overlap with the thyroid hormone measurement subsamples in NHANES.

To assess disease specificity, associations were examined between PFOA and the other NHANES disease categories elicited: ischemic heart disease (combining any diagnoses of coronary heart disease, angina, and/or heart attack), diabetes, arthritis, current asthma, chronic obstructive pulmonary disease (COPD; bronchitis or emphysema), and current liver disease.

### Statistical analysis

NHANES uses a complex cluster sample design with some demographic groups (including less-privileged socioeconomic groups and Mexican Americans) oversampled to ensure adequate representation. Prevalence estimates and models were therefore survey-weighted using the NHANES primary sampling unit, strata, and population weights, unless otherwise stated.

Multivariate logistic regression modeling was used to estimate odds ratios (ORs) of thyroid disease outcomes by quartile of PFOA and PFOS concentrations, and associations of other physician-diagnosed diseases. Because thyroid disease prevalence is markedly higher in women, we used sex-specific models. Because the distribution of PFC concentrations is skewed (with most people having relatively low exposures and with considerably more variance at the higher exposure end), all available data were pooled, and PFOA and PFOS concentrations were divided into population-weighted quartiles. Using the Hsieh method (Hsieh et al. 1998), our estimated power to detect an association of OR ≥ 1.8 with current treated disease comparing the top PFOA quartile (Q4) with bottom quartile (Q1) is 67% in women. Combining the lowest two quartiles (Q1 and Q2) into a larger control group provides 80% power. The corresponding minimum detectable effect size in men is OR > 2.9. Assumptions for the power calculations include a significance level of 5% and a multiple correlation coefficient of 0.2 relating PFOA exposure to potential confounders.

Models were adjusted for the following potential confounding factors: year of NHANES study; age; sex; race/ethnicity, from self-description and categorized into Mexican American, other Hispanic, non-Hispanic white, non-Hispanic black, and other race (including multiracial); education, categorized into less than high school, high school diploma (including GED), more than high school, and unknown education; smoking (from self-reported status asked for those ≥ 20 years of age), categorized into never smoked, former smoker, smoking some days, smoking every day, and unknown smoking status; body mass index (BMI; weight in kilograms divided by the square of measured height in meters), categorized into underweight (BMI < 18.5), recommended weight (BMI = 18.5–24.9), overweight (BMI = 25.0–29.9), obese (BMI = 30.0–34.9), and unknown BMI; and alcohol consumption (in adults ≥ 20 years of age, based on responses to the question “In the past 12 months, on those days that you drank alcoholic beverages, on the average day, how many drinks did you have?”), categorized into 0, 1, 2, 3, 4, and ≥ 5 drinks per day, and unknown alcohol consumption. Regression analyses were conducted using STATA/SE (version 10.1; StataCorp LP, College Station, TX, USA).

## Results

Serum concentrations of PFOA were available for *n* = 3,974 individuals ≥ 20 years of age from NHANES waves 1999–2000 (*n* = 1,040), 2003–2004 (*n* = 1,454), and 2005–2006 (*n* = 1,480). In analyses adjusted for age, sex, NHANES wave, and ethnicity, mean levels of PFOA were higher in men than in women [by 0.76 ng/mL; 95% confidence interval (CI), 0.73–0.80; *p* < 0.0001], and we found significant differences between ethnic groups (Table 1). Individuals with more education had higher PFOA levels (highest vs. lowest education: 1.1 ng/mL difference; 95% CI, 1.03–1.19 ng/mL difference; *p* = 0.008). Increased alcohol consumption levels were also associated with higher PFOA concentrations (e.g., those having five or more drinks per day had mean PFOA levels 1.24 ng/mL higher than nondrinkers; 95% CI, 1.14–1.37 ng/mL difference; *p* < 0.0001).

In analyses of the full sample (men and women), adjusted for age, sex, NHANES wave, and ethnicity, mean levels of PFOA were higher in men than in women (*p* < 0.0001), and we found significant differences among ethnic groups (Table 1). Individuals with more education had higher PFOA levels (*p* = 0.008). Increased alcohol consumption levels were also associated with higher PFOA concentrations (*p* < 0.0001). We found similar differences in PFOS concentrations. Mean levels of PFOS were higher in men (*p* < 0.0001), with significant differences in levels between ethnic groups, and individuals with more education had higher PFOS levels (*p* = 0.008).

Eight individuals did not answer questions about thyroid disease, so the sample size for this analysis was *n* = 3,966, with 1,900 men, and 2,066 women (Table 2). In women, overall (unweighted) reported (any) thyroid disease was *n* = 292, and the NHANES-weighted but unadjusted prevalence was 16.18%; in men, *n* = 69, and weighted prevalence, 3.06%. The study-weighted prevalence of current thyroid disease taking medication was necessarily lower (women, *n* = 163, 9.89%; men, *n* = 46, 1.18%).

We computed population-weighted quartiles of PFOA and PFOS concentrations in men and women separately (Table 2). The highest quartile (Q4) of PFOA in women ranged from 5.7 to 123.0 ng/mL, and in men from 7.3 ng/mL to 45.9 ng/mL. Study-weighted but unadjusted prevalences of current thyroid disease taking related medication in women varied across the quartiles but with wide CIs: Q1 = 8.14% (95% CI, 5.75–10.53%), Q4 = 16.19% (95% CI, 11.74–20.62%); in men, unadjusted prevalence rates were far lower throughout (prevalence = 2.27% in Q1 and Q4). For PFOS the prevalence of treated thyroid disease ranged from 8.14% (Q1) to 12.55% (Q4) in women and from 1.85% to 3.89% in men.

In logistic regression models adjusting for age, ethnicity, and study year (Table 3), we found associations between PFOA quartiles and both definitions of thyroid disease in women. For logistic models additionally adjusted for educational status, BMI, smoking status, and alcohol consumption, these associations remained significant; for example, comparing those with PFOA concentrations ≥ 5.7 ng/mL (Q4) versus ≤ 2.6 ng/mL (Q1), the OR for current thyroid disease on medication was 1.86 (95% CI, 1.12–3.09; *p* = 0.018). Comparing Q4 with the larger control group of PFOA ≤ 4.0 ng/mL (Q1 and Q2) the estimated OR for treated thyroid disease was 2.24 (95% CI, 1.38–3.65; *p* = 0.002). In men, we found a similar suggestive trend, but it narrowly missed significance: Comparing PFOA concentrations ≥ 7.3 ng/mL (Q4) versus ≤ 5.2 ng/mL (Q1 and Q2), the OR for treated disease was 2.12 (95% CI, 0.93–4.82; *p* = 0.073).

For PFOS concentrations, in women ORs for disease trended in a similar direction but were far from significant. However, in men we found an association comparing those with PFOS concentrations ≥ 36.8 ng/mL (Q4) versus ≤ 25.5 ng/mL (Q1 and Q2): OR for treated disease was 2.68 (95% CI, 1.03–6.98; *p* = 0.043).

### Sensitivity analyses

For a sensitivity analysis, we computed a logistic regression model including both men and women, testing an interaction term between sex and PFOA levels for treated thyroid disease risk. The interaction term was not significant (*p*-value for interaction = 0.152).

For a post hoc analysis, we examined associations between chemical concentration quartile and any of the other major disease categories covered in NHANES: arthritis, asthma, COPD, diabetes, heart disease, or liver disease. Combining men and women (to reduce multiple testing and because these diseases are less sex related), we found no significant associations for PFOA (Table 4) except for comparisons between the intermediate quartiles and Q1 for arthritis, but this did not reach significance in the top quartile.

For PFOS, we found no “positive” associations between higher serum concentrations and higher prevalence of disease. We found one statistically significant “negative” association suggesting that people reporting having COPD may be less likely to be in the highest PFOA concentration quartile (OR = 0.58; 95% CI, 0.43–0.76; *p* = 0.0003).

## Discussion

In this study we aimed to determine whether increased serum PFOA or PFOS concentrations were associated with thyroid disease in a general adult U.S. population sample. The prevalence of thyroid disease is markedly higher in women than in men, so we estimated sex-specific associations. We found that, across all the available data from NHANES, thyroid disease associations with serum PFOA concentrations are present in women and are strongest for those currently being treated for thyroid disease. In men, we also found a near significant association between PFOA and treated thyroid disease. An interaction term analysis suggests that the PFOA trends in men and women are not significantly different, despite the relative rarity of thyroid disease in men. In addition, we found a nominally significant association between PFOS concentrations and treated thyroid disease in men but not in women.

The presence of associations with both PFOA and PFOS raises the issue of how best to perform risk assessments for combinations of perfluorochemicals. The somewhat divergent risk patterns for the two compounds support their separate risk assessment (Scialli et al. 2007), given that current legislative advice (Minnesota Department of Health 2008) is to consider the combined effects of chemicals only when two or more chemicals in a mixture affect the same tissue, organ, or organ system.

Our results are important because PFAAs are detectable in virtually everyone in society (Kannan et al. 2004), with ubiquitous presence across global populations (Calafat et al. 2006). Occupational exposure to PFOA reported in 2003 showed mean serum values of 1,780 ng/mL (range, 40–10,060 ng/mL) (Olsen et al. 2003a) and 899 ng/mL (range, 722–1,120 ng/mL) (Olsen et al. 2003c). Production of PFOS was halted in 2002 in the United States by its principal producer, due largely to concerns over bioaccumulation and toxicity. Since then, voluntary industry reductions in production and use of other perfluorinated compounds, such as the U.S. EPA–initiated PFOA Stewardship Program (U.S. EPA 2006), have contributed to a decreasing trend in human exposure for all perfluorinated compounds (with the notable exception of perfluorononanoic acid) (Calafat et al. 2007; Olsen et al. 2007). In May 2009, PFOS was listed under the Stockholm Convention on Persistent Organic Pollutants (2008).

Our results can be compared with previous studies of human populations and of nonhuman primates. A 6-month study of cynomolgus monkeys chronically exposed to PFOA showed no associations between PFOA and thyroid parameters, at mean serum PFOA concentrations higher than those reported in NHANES, although only male monkeys were involved (Butenhoff et al. 2002). The largest human study of PFOA centers on an industrial facility in Washington, West Virginia, from which PFOA spread to the population through air, water, occupational, and domestic exposure in a point-source contamination. The C8 Health Project (Steenland et al. 2009) has measured PFOA concentrations in > 69,000 residents. Markedly high concentrations were found, with an arithmetic mean of 83 ng/mL and a median concentration in serum of 28 ng/mL (C8 Science Panel 2008), far higher than the NHANES concentrations in the general population. Preliminary analyses report associations between PFOA and total cholesterol, low-density lipoproteins, and triglyceride concentrations in multivariate models adjusting for age, BMI, sex, education, smoking, alcohol, and regular exercise. Comprehensive cross-sectional and follow-on analyses of associations with thyroid disease have not yet been reported but are expected to be released in 2010–2011 (C8 Science Panel 2009).

Importantly, disruption to thyroid hormone balance was not found in other studies of populations exposed to PFOA, despite the considerably higher levels reported in some studies (Emmett et al. 2006; Olsen et al. 2003b). Emmett et al. (2006) studied 371 residents of a community with long-standing environmental exposure to PFOA and found a median serum PFOA concentration of 181–571 ng/mL but no association between serum PFOA and a history of thyroid disease. A study that included thyroid hormone levels reported a positive association between serum PFOA concentration and T_3_ levels in occupationally exposed workers, although there were no changes in other thyroid hormones (Olsen et al. 2001). Modest associations between PFOA and thyroid hormones (negative for free T_4_ and positive for T_3_) were reported in 506 PFOA production workers across three production facilities (Olsen and Zobel 2007); there were no associations between TSH or T_4_ and PFOA, and the free hormone levels were within the normal reference range.

A linear extrapolation of the findings reported here would be expected to lead to associations being more evident at higher exposure levels, yet this is not supported by the literature. Nonlinearity of response is not uncommon for receptor-mediated systems such as endocrine-signaling pathways that act to amplify the original signal. Large changes in cell function can occur in response to extremely low concentrations, but which may become saturated and hence unresponsive at higher concentrations (vom Saal and Hughes 2005; Welshons et al. 2003).

The mechanisms involved in thyroid homeostasis are numerous and complex, and there are multiple potential targets for disruption of thyroid hormone homeostasis (Schmutzler et al. 2007). These include thyrotropin receptor (Santini et al. 2003), iodine uptake by the sodium iodide transporter (Schröder van der Elst et al. 2003), type 1 5′-deiodinase (Ferreira et al. 2002), transthyretin (Kohrle et al. 1988), thyroid hormone receptor (Moriyama et al. 2002), and the thyroid hormone–dependent growth of pituitary cells (Ghisari and Bonefeld-Jorgensen 2005). Depression of serum T_4_ and T_3_ has been reported by several authors in PFOS-exposed rats (Lau et al. 2003; Luebker et al. 2005; Seacat et al. 2003). One mechanism by which PFAAs may deplete T_4_ is through induction of the hepatic uridine diphosphoglucuronosyl transferase (UGT) system, which is involved in hepatic metabolism of thyroid hormone and biliary clearance of T_4_ as T_4_-glucuronide (Barter and Klaassen 1994). Because PFOA is an agonist for PPARα, it is plausible that induction of hepatic UGT in PFAA-exposed rats (Yu et al. 2009) could represent a PPARα-mediated response. The involvement of another PPARα agonist, WY 14643, in enhancing the hepatic degradation of thyroid hormone has recently been shown (Weineke et al. 2009).

A growing body of data describes the *in vitro* binding affinity of PFOA to human serum-binding proteins (Chen and Guo 2009) and to PPARα, -β, and -γ and other nuclear receptors (Vanden Huevel et al. 2006), but the contribution of these mechanisms to PFOA’s thyroid-mediating effects in humans remains to be established. Many cellular and metabolic processes, including lipid metabolism, energy homeostasis, and cell differentiation, are controlled by PPARα. Early studies of the effects of PFAAs in rodents showed that a single dose lowered heart rate and body temperature and depressed T_4_ and T_3_. Replacement of T_4_ did not reverse the clinical symptoms of hypothermia (Gutshall et al. 1988; Langley and Pilcher 1985). Although circulating thyroid hormone levels were low, liver enzymes responsive to thyroid hormone levels were elevated, suggesting that thyroidal homeostasis was not functionally compromised. Chang et al. (2007) found that exposure to PFOS for up to 3 weeks did not affect functional thyroid status, because free T_4_, TSH, and various thyroid-responsive liver enzymes were all unaffected. These findings and later results have led to proposals that displacement of circulating thyroid hormones from plasma protein-binding sites and a reduced responsiveness of the HPT axis contribute significantly towards PFOA’s hypothyroid-inducing effects (Lau et al. 2007). Whatever the mechanisms involved, it is clear that more research is merited to clarify the pathways involved.

The feedback mechanism by which the rate of release of TSH and the circulating levels of T_3_ and T_4_ are regulated tends to show a low level of individual variation (Felt-Rasmussen et al. 1980). Therefore, subtle disruption of the HPT axis within normal reference ranges may have negative health consequences for the individual while remaining within normal reference values, highlighting the importance of including both clinical and laboratory end points in such studies. The NHANES data do not allow specification of the precise type of thyroid disease present, because NHANES does not report on individual hormone levels. PFOA concentration was positively associated with free T_4_ and negatively associated with T_3_ levels in a cohort of 506 exposed workers, with a near significant association with TSH levels (Olsen et al. 2007), although all effects were regarded as modest.

The limitations of these analyses should be noted. We based the PFOA and PFOS measures on a single serum sample. Although PFOA has a half-life of 4 years (Olsen et al. 2007), so a single sample is likely to represent medium-term internal dose, samples taken at several time points might be more accurate in classifying exposure. Any misclassification from single measures would tend to decrease power and underestimate the real strengths of association. Second, the PFOA concentrations were measured at the same time as disease status, making attribution of causal direction difficult. This raises the possibility of reverse causation. One might hypothesize that after onset of thyroid disease, changes in the nature of exposure or in the pharmacokinetics of PFOA might occur [including patterns of absorption, distribution (including protein binding) or excretion]. Because the associations we report were present in people who were on thyroid hormone replacements, which effectively mimic normal thyroid function, a mechanism for reverse causation through changes in pharmacokinetics is difficult to imagine. Confounding by unmeasured factors is also possible, but it is unlikely that confounding could explain similar findings reported from some of the diverse experimental and observational studies discussed above.

Post hoc association testing with other common diseases (necessarily involving multiple statistical testing) did not identify other robust associations of higher PFC concentration with increased disease prevalence, suggesting specificity of our findings for thyroid disease. An apparent association between higher PFOS concentrations and lower prevalence of COPD requires replication, to exclude a false-positive result from multiple testing.

In addition to the limitations of our analyses, the strengths should also be noted: This is the first large-scale analysis in a nationally representative general adult population of directly measured serum concentrations of PFOA and PFOS. In addition, the associations present are strongest for the most specific identification of thyroid disease, based on reported diagnosis with current use of thyroid-specific medication. The NHANES study also supported adjustment of models for a range of potential confounding factors, which in fact made relatively minor differences to the key estimates, suggesting that the associations are robust.

Further work is clearly needed to characterize the PFOA and PFOS associations with specific thyroid diagnoses and thyroid hormone levels in the general population and to clarify whether the associations reflect pathology, changes in exposure, or altered pharmacokinetics. Longitudinal analyses are also needed to establish whether high exposures predict future onsets of thyroid disease, although concurrent alteration of thyroid functioning would still be a cause for concern.

## Conclusions

Higher PFOA and PFOS concentrations are associated with thyroid disease (and being on thyroid-related medication) in the NHANES U.S. general adult population representative study samples. More work is needed to establish the mechanisms underlying this association and to exclude confounding and pharmacokinetic explanations.

## Figures and Tables

**Table 1:** Survey-weighted characteristics of sample with survey-weighted back-transformed geometric mean concentrations (95% CIs) of PFOA and PFOS.

	Men			Women		
Characteristic	*n* (% within group)	PFOA	PFOS	*n* (% within group)	PFOA	PFOS
Overall	1,900 (100)	4.91 (4.64–5.2)	25.08 (23.63–26.62)	2,066 (100)	3.77 (3.52–4.04)	19.14 (17.8–20.58)

Age (years)						
20–49	928 (62.6)	5.30 (5.02–5.59)	24.2 (22.66–25.84)	1,134 (59.8)	3.38 (3.13–3.64)	16.72 (15.37–18.19)
50–69	553 (27.0)	4.46 (4.12–4.82)	26.97 (24.87–29.25)	545 (26.7)	4.62 (4.15–5.14)	22.92 (20.81–25.23)
≥ 70	419 (10.4)	3.99 (3.53–4.52)	25.8 (23.22–28.67)	387 (13.4)	4.13 (3.78–4.51)	24.39 (22.19–26.81)

Ethnicity						
Mexican American	432 (8.3)	3.73 (3.48–4)	18.44 (16.79–20.25)	481 (7.3)	2.44 (2.21–2.7)	12.04 (10.91–13.29)
Other Hispanic	62 (4.2)	5.63 (4.77–6.65)	27.73 (21.89–35.14)	92 (5.9)	3.6 (3.17–4.09)	16.28 (14.29–18.56)
Non-Hispanic white	969 (73.5)	5.13 (4.83–5.45)	25.7 (24.04–27.47)	1,008 (71.8)	4.13 (3.84–4.45)	20.08 (18.57–21.72)
Non-Hispanic black	382 (9.9)	4.43 (3.92–5)	27 (24.3–30)	415 (10.8)	2.98 (2.66–3.34)	20.52 (17.97–23.42)
Other	55 (4.1)	4.32 (3.47–5.38)	22.77 (17.3–29.97)	70 (4.3)	3.3 (2.61–4.17)	19.64 (15.04–25.64)

Education						
< High school	637 (20.9)	4.14 (3.82–4.49)	22.08 (20.47–23.82)	628 (19.5)	3.53 (3.11–4)	19.26 (17.1–21.69)
High school graduate	478 (29.3)	4.94 (4.56–5.35)	25.22 (23.56–27)	498 (25.9)	4 (3.69–4.34)	19.9 (18.15–21.83)
> High school	782 (49.7)	5.26 (4.93–5.62)	26.38 (24.54–28.36)	937 (54.6)	3.76 (3.49–4.04)	18.74 (17.32–20.28)
Unknown	3 (0.0)	2.22 (1.31–3.74)	17.59 (8.5–36.41)	3 (0.0)	1.84 (1.32–2.56)	12.64 (7.25–22.02)

BMI						
0–18.5	23 (1.2)	4.42 (2.78–7.01)	17.95 (10.79–29.87)	35 (2.4)	3.78 (3.07–4.65)	18.39 (14.05–24.08)
18.5–25	525 (28.8)	4.89 (4.44–5.39)	24.36 (21.99–26.98)	594 (32.6)	3.84 (3.52–4.19)	18.97 (17.53–20.52)
25–30	778 (39.8)	5.01 (4.67–5.38)	25.28 (23.55–27.13)	615 (27.8)	3.64 (3.3–4.01)	19.48 (17.86–21.24)
≥ 30	545 (29.0)	4.9 (4.54–5.29)	26.28 (24.42–28.28)	784 (36.0)	3.83 (3.49–4.2)	19 (17–21.22)
Unknown	29 (1.2)	3.1 (2.23–4.3)	17.51 (10.34–29.66)	38 (1.2)	3.36 (2.78–4.05)	21.81 (17.13–27.78)

Smoking status						
Smoked < 100 cigarettes in lifetime	761 (41.1)	5.01 (4.68–5.37)	26.16 (24.19–28.29)	1,300 (58.6)	3.63 (3.36–3.93)	18.91 (17.43–20.52)
Former smoker	664 (30.0)	4.67 (4.28–5.09)	25.66 (23.7–27.79)	417 (21.3)	3.81 (3.46–4.19)	19.22 (17.33–21.32)
Some days	90 (4.8)	5.12 (4.23–6.19)	21.33 (17.97–25.31)	46 (2.4)	4.05 (3.36–4.89)	18.29 (15.06–22.22)
Every day	385 (24.1)	5.01 (4.7–5.33)	23.43 (21.45–25.59)	302 (17.8)	4.17 (3.73–4.67)	19.9 (17.76–22.29)
Unknown				1 (0.0)		

Average drinks per day in previous 12 months						
Nondrinker	334 (15.8)	4.44 (3.89–5.06)	25.38 (22.78–28.28)	857 (35.1)	3.5 (3.22–3.8)	18.84 (16.81–21.11)
1	298 (15.7)	4.67 (4.22–5.17)	26.39 (23.19–30.02)	316 (17.9)	3.87 (3.47–4.32)	20.37 (18.12–22.9)
2	282 (17.5)	5.4 (4.86–6.01)	27.7 (24.76–30.99)	291 (18.0)	4.15 (3.79–4.55)	19.82 (17.95–21.87)
3	181 (11.1)	5.2 (4.79–5.65)	23.73 (21.2–26.56)	124 (6.6)	3.82 (3.4–4.29)	18.07 (15.81–20.65)
4	97 (6.0)	5.58 (4.94–6.31)	25.05 (21.27–29.5)	64 (3.3)	3.96 (3.19–4.92)	15.71 (12.89–19.14)
≥ 5	283 (16.4)	5.25 (4.88–5.65)	23.09 (20.92–25.49)	80 (4.8)	4.63 (3.62–5.91)	18.13 (14.76–22.27)
Unknown	425 (17.6)	4.44 (4.07–4.84)	24.04 (21.44–26.96)	334 (14.4)	3.58 (3.22–3.98)	19.26 (17.29–21.46)

**Table 2:** Sex-specific summary statistics with thyroid disease prevalence by weighted (95% CIs) quartile of PFOA and PFOS.

	Summary statistics by quartile				Thyroid disease ever			Current thyroid disease with thyroid medication		
	*n*	Range (ng/mL)	Mean ± SD (ng/mL)	Weighted mean (95% CI) (ng/mL)	*n* (case/total)	Prevalence (%)		*n* (case/total)	Prevalence (%)	
						Unweighted	Weighted		Unweighted	Weighted
Women										
PFOA										
All	2,066	0.1–123.0	4.25 ± 4.92	4.84 (4.35–5.33)	292/2,066	14.13	16.18 (14.44–18.09)	163/2,066	7.89	9.89 (8.32–11.72)
Q1	689	0.1–2.6	1.71 ± 0.66	1.79 (1.72–1.85)	65/689	9.43	12.62 (9.66–15.57)	34/689	4.93	8.14 (5.75–10.53)
Q2	550	2.7–4.0	3.32 ± 0.40	3.33 (3.28–3.38)	71/550	12.91	13.87 (9.66–18.08)	35/550	6.36	7.27 (4.37–10.16)
Q3	441	4.1–5.7	4.79 ± 0.48	4.78 (4.72–4.85)	72/441	16.33	15.98 (11.81–20.15)	39/441	8.84	8.25 (4.97–11.52)
Q4	386	5.7–123.0	9.47 ± 9.38	9.7 (8.43–10.98)	84/386	21.76	22.57 (17.44–27.71)	55/386	14.25	16.18 (11.74–20.62)

PFOS										
All	2,066	0.14–406.0	23.24 ± 23.13	24.78 (22.6–26.9)	292/2,066	14.13	16.18 (14.44–18.09)	163/2,066	7.89	9.89 (8.32–11.72)
Q1	616	0.14–12.4	8.13 ± 2.82	8.49 (8.06–8.93)	68/616	11.04	15.14 (10.82–19.46)	32/616	5.19	8.7 (5.45–11.96)
Q2	523	12.5–19.4	15.75 ± 2.02	15.92 (15.71–16.13)	71/523	13.58	16.23 (12.58–19.89)	40/523	7.65	9.85 (6.58–13.13)
Q3	466	19.5–29.8	24.21 ± 2.89	24.49 (24.03–24.94)	62/466	13.30	12.69 (8.59–16.78)	39/466	8.37	8.47 (4.98–11.97)
Q4	461	29.9–406.0	50.96 ± 35.15	50.48 (45.81–55.16)	91/461	19.74	20.66 (16.46–24.87)	52/461	11.28	12.55 (8.86–16.23)

Men										
PFOA										
All	1,900	0.1–45.9	5.23 ± 3.41	5.79 (5.41–6.18)	69/1,900	3.63	3.06 (2.40–3.88)	46/1,900	2.42	1.88 (1.30–2.69)
Q1	643	0.1–3.6	2.47 ± 0.85	2.51 (2.43–2.58)	24/643	3.73	3.49 (2.01–4.97)	16/643	2.49	2.27 (1.25–3.30)
Q2	517	3.7–5.2	4.42 ± 0.45	4.44 (4.39–4.50)	20/517	3.87	3.44 (1.48–5.41)	13/517	2.51	2.14 (0.79–3.49)
Q3	381	5.3–7.2	6.12 ± 0.55	6.19 (6.12–6.26)	11/381	2.89	1.51 (0.38–2.63)	7/381	1.84	0.77 (0.17–1.37)
Q4	359	7.3–45.9	10.39 ± 4.20	10.3 (9.72–10.89)	14/359	3.90	3.71 (1.67–5.75)	10/359	2.79	2.27 (0.22–4.33)

PFOS										
All	1,900	0.3–435.0	29.57 ± 22.11	30.36 (28.2–32.5)	69/1,900	3.63	3.06 (2.40–3.88)	46/1,900	2.42	1.88 (1.30–2.69)
Q1	529	0.3–18.0	12.29 ± 4.30	12.35 (11.94–12.76)	18/529	3.40	3.22 (1.86–4.57)	10/529	1.89	1.85 (0.82–2.89)
Q2	480	18.2–25.5	21.82 ± 2.13	21.83 (21.63–22.03)	13/480	2.71	1.64 (0.40–2.87)	8/480	1.67	0.80 (0.12–1.48)
Q3	454	25.6–36.7	30.81 ± 3.18	30.93 (30.57–31.29)	15/454	3.30	2.68 (1.26–4.10)	11/454	2.42	1.62 (0.55–2.69)
Q4	437	36.8–435.0	57.73 ± 29.4	56.45 (52.85–60.04)	23/437	5.26	4.69 (2.44–6.95)	17/437	3.89	3.24 (1.07–5.40)

Quartiles (Q1–Q4) reflect the U.S. population, accounting for population weighting in NHANES.

**Table 3:** Sex-specific, survey-weighted associations between PFOA and PFOS concentrations and thyroid disease [OR (95% CI), *p*-value].

	PFOA		PFOS	
Group/quartile	Models adjusting for age, ethnicity, and study year	Fully adjusted models*a*	Models adjusting for age, ethnicity, and study year	Fully adjusted models*a*
Women				
Thyroid disease ever				
Q1	1	1	1	1
Q2	0.98 (0.65–1.50), *p* = 0.936	0.95 (0.62–1.47), *p* = 0.825	1.04 (0.63–1.71), *p* = 0.875	1.01 (0.63–1.6), *p* = 0.972
Q3	1.09 (0.66–1.81), *p* = 0.729	1.11 (0.67–1.83), *p* = 0.679	0.68 (0.4–1.17), *p* = 0.155	0.64 (0.39–1.05), *p* = 0.078
Q4	1.63 (1.07–2.47), *p* = 0.024*	1.64 (1.09–2.46), *p* = 0.019*	1.11 (0.66–1.86), *p* = 0.69	1.15 (0.7–1.91), *p* = 0.568
Q4 vs. Q1 and Q2	1.64 (1.12–2.41), *p* = 0.013*	1.68 (1.14–2.49), *p* = 0.011*	1.08 (0.73–1.61), *p* = 0.681	1.15 (0.78–1.7), *p* = 0.48

Thyroid disease current with medication				
Q1	1	1	1	1
Q2	0.77 (0.45–1.32), *p* = 0.334	0.7 (0.41–1.22), *p* = 0.205	1.11 (0.58–2.14), *p* = 0.747	1.05 (0.55–2), *p* = 0.89
Q3	0.86 (0.47–1.57), *p* = 0.607	0.89 (0.49–1.59), *p* = 0.676	0.85 (0.46–1.59), *p* = 0.609	0.81 (0.44–1.51), *p* = 0.496
Q4	1.83 (1.13–2.95), *p* = 0.015*	1.86 (1.12–3.09), *p* = 0.018*	1.27 (0.69–2.32), *p* = 0.435	1.31 (0.72–2.36), *p* = 0.369
Q4 vs. Q1 and Q2	2.09 (1.34–3.26), *p* = 0.002*	2.24 (1.38–3.65), *p* = 0.002*	1.19 (0.77–1.85), *p* = 0.417	1.27 (0.82–1.97), *p* = 0.269

Men				
Thyroid disease ever				
Q1	1	1	1	1
Q2	1.17 (0.64–2.15), *p* = 0.600	1.11 (0.62–1.99), *p* = 0.729	0.50 (0.22–1.17), *p* = 0.107	0.51 (0.23–1.14), *p* = 0.097
Q3	0.58 (0.21–1.59), *p* = 0.283	0.57 (0.19–1.66), *p* = 0.291	0.81 (0.40–1.61), *p* = 0.536	0.88 (0.43–1.84), *p* = 0.736
Q4	1.58 (0.79–3.16), *p* = 0.191	1.58 (0.74–3.39), *p* = 0.233	1.51 (0.70–3.22), *p* = 0.284	1.58 (0.72–3.47), *p* = 0.251
Q4 vs. Q1 and Q2	1.45 (0.68–3.09), *p* = 0.323	1.5 (0.66–3.39), *p* = 0.324	1.6 (0.57–4.46), *p* = 0.360	1.78 (0.58–5.52), *p* = 0.309

Thyroid disease current with medication				
Q1	1	1	1	1
Q2	1.18 (0.55–2.54), *p* = 0.668	1.12 (0.52–2.39), *p* = 0.767	0.42 (0.16–1.10), *p* = 0.077	0.43 (0.17–1.08), *p* = 0.073
Q3	0.51 (0.20–1.32), *p* = 0.162	0.49 (0.18–1.38), *p* = 0.171	0.82 (0.29–2.27), *p* = 0.694	0.95 (0.34–2.70), *p* = 0.926
Q4	1.74 (0.63–4.78), *p* = 0.275	1.89 (0.60–5.90), *p* = 0.268	1.72 (0.73–4.05), *p* = 0.211	1.89 (0.72–4.93), *p* = 0.190
Q4 vs. Q1 and Q2	2.02 (0.89–4.58), *p* = 0.092	2.12 (0.93–4.82), *p* = 0.073	2.44 (1.04–5.74), *p* = 0.041*	2.68 (1.03–6.98), *p* = 0.043*

a Models adjusted for age, ethnicity, education, BMI, smoking status, and alcohol consumption.

* *p* < 0.05.

**Table 4:** Associations between PFOA and PFOS concentrations (population-based quartiles) and other diseases in fully adjusted logistic regression models (by self-reported disease status).

	PFOA			PFOS		
Disease/quartile	*n* (% survey-weighted)	OR (95% CI)	*p*-Value	*n* (% survey-weighted)	OR (95% CI)	*p*-Value
Arthritis ever	1,006/3,960 (22.8)			1,006/3,960 (22.8)		

Q1	287/1,310 (19.2)	1	1	219/1,132 (19.0)	1	1
Q2	298/1,036 (27.6)	1.63 (1.24–2.14)	0.001	267/1,009 (23.5)	1.19 (0.91–1.54)	0.193
Q3	231/857 (22.7)	1.31 (1.03–1.66)	0.029	260/916 (26.8)	1.29 (1.00–1.66)	0.054
Q4	190/757 (21.8)	1.28 (0.97–1.68)	0.082	260/903 (22.0)	0.74 (0.53–1.04)	0.085

Asthma ever	471/3,961 (13.2)			471/3,961 (13.2)		

Q1	138/1,313 (11.9)	1	1	139/1,133 (14.0)	1	1
Q2	128/1,036 (14.2)	1.25 (0.92–1.70)	0.154	140/1,013 (15.6)	1.16 (0.80–1.68)	0.427
Q3	122/856 (15.8)	1.44 (1.01–2.05)	0.045	111/914 (13.1)	0.97 (0.65–1.43)	0.867
Q4	83/756 (11.2)	0.93 (0.64–1.36)	0.716	81/901 (10.3)	0.79 (0.50–1.26)	0.320

COPD ever	302/3,953 (8.2)			302/3,953 (8.2)		

Q1	81/1,310 (7.7)	1	1	83/1,131 (8.8)	1	1
Q2	93/1,033 (8.8)	0.91 (0.58–1.43)	0.677	85/1,008 (8.5)	0.84 (0.56–1.25)	0.384
Q3	66/853 (8.3)	0.88 (0.54–1.43)	0.593	67/914 (7.7)	0.67 (0.41–1.09)	0.103
Q4	62/757 (8.2)	0.85 (0.54–1.34)	0.473	67/900 (7.9)	0.58 (0.43–0.76)	0.0003

Diabetes ever	459/3,964 (8.7)			459/3,964 (8.7)		

Q1	186/1,314 (10.9)	1	1	122/1,133 (8.6)	1	1
Q2	127/1,035 (9.2)	0.80 (0.55–1.17)	0.242	119/1,012 (9.3)	1.02 (0.70–1.47)	0.928
Q3	83/857 (7.7)	0.74 (0.48–1.15)	0.177	103/916 (7.7)	0.76 (0.50–1.18)	0.218
Q4	63/758 (7.0)	0.69 (0.41–1.16)	0.158	115/903 (9.4)	0.87 (0.57–1.31)	0.491

Heart disease ever*a*	321/3,966 (5.8)			321/3,966 (5.8)		

Q1	93/1,314 (5.7)	1	1	69/1,134 (4.8)	1	1
Q2	93/1,037 (6.1)	0.95 (0.59–1.51)	0.816	85/1,013 (5.1)	0.77 (0.49–1.23)	0.270
Q3	78/857 (5.9)	1.02 (0.65–1.61)	0.917	80/916 (5.7)	0.83 (0.46–1.51)	0.540
Q4	57/758 (5.4)	1.08 (0.70–1.69)	0.715	87/903 (7.4)	0.91 (0.50–1.64)	0.745

Liver disease current	57/3,942 (1.4)			57/3,942 (1.4)		

Q1	24/1,307 (1.4)	1	1	22/1,127 (1.7)	1	1
Q2	11/1,028 (1.0)	0.66 (0.25–1.74)	0.391	10/1,007 (0.9)	0.49 (0.18–1.32)	0.154
Q3	17/855 (2.5)	1.93 (0.96–3.88)	0.065	13/910 (1.6)	0.94 (0.41–2.16)	0.880
Q4	5/752 (0.8)	0.61 (0.21–1.78)	0.355	12/898 (1.5)	0.95 (0.39–2.29)	0.907

a Any report of coronary heart disease, and/or angina, and/or heart attack.

## References

[b1-ehp-118-686] Barter R, Klaassen C (1994). Reduction of thyroid hormone levels and alteration of thyroid function by four representative UDP-glucuronosyl transferase inducers in rats. Toxicol Appl Pharmacol.

[b2-ehp-118-686] Boas M, Feldt-Rasmussen U, Skakkebaek N, Main K (2006). Environmental chemicals and thyroid function. Eur J Endocrinol.

[b3-ehp-118-686] Butenhoff J, Costa G, Elcombe C, Farrar D, Hansen K, Iwai H (2002). Toxicity of ammonium perfluorooctanoate in male cynomolgus monkeys after oral dosing for 6 months. Toxicol Sci.

[b4-ehp-118-686] Calafat AM, Kuklenyik Z, Caudill SP, Reidy JA, Needham LL (2006). Perfluorochemicals in pooled serum samples from United States residents in 2001 and 2002. Environ Sci Technol.

[b5-ehp-118-686] Calafat AM, Wong LY, Kuklenyik Z, Reidy JA, Needham LL (2007). Polyfluoroalkyl chemicals in the U.S. population: data from the National Health and Nutrition Examination Survey (NHANES) 2003–2004 and comparisons with NHANES 1999–2000. Environ Health Perspect.

[b6-ehp-118-686] C8 Science Panel (2009). Cross Sectional Study of C8 and Immune Function, Hematopoietic Function, Liver, Kidney, and Endocrine Disorders and Cancer Prevalence—a Prevalence Study among Participants in the C8 Health Project [abstract]. http://www.C8sciencepanel.org/studies.html#2.

[b7-ehp-118-686] C8 Science Panel (2008). Status Report: Factors Associated with PFOA Levels in a Community Surrounding a Chemical Plant. http://c8sciencepanel.org/pdfs/Status_Report-factors_associated_with_C8_levels_Oct2008.pdf.

[b8-ehp-118-686] Chang S, Hart J, Ehresman D, Das K, Lau C, Noke P (2007). The pharmacokinetics of perfluorobutyrate in rats, mice and monkeys. Toxicologist.

[b9-ehp-118-686] Chen Y, Guo L (2009). Fluorescence study on site-specific binding of perfluoroalkyl acids to human serum albumin. Arch Toxicol.

[b10-ehp-118-686] Emmett EA, Zhang H, Shofer FS, Freeman D, Rodway NV, Desai C (2006). Community exposure to perfluorooctanoate: relationships between serum levels and certain health parameters. J Occup Environ Med.

[b11-ehp-118-686] Felt-Rasmussen U, Hyltoft P, Blaabjerg O, Horder M (1980). Long term variability in serum thyroglobulin and thyroid related hormones in healthy subjects. Acta Endocrinol (Copenh).

[b12-ehp-118-686] Ferreira AC, Lisboa PC, Oliveira KJ, Lima LP, Barros IA, Carvalho DP (2002). Inhibition of thyroid type 1 deiodinase activity by flavonoids. Food Chem Toxicol.

[b13-ehp-118-686] Fromme H, Tittlemier SA, Volkel W, Wilhelm M, Twardella D (2009). Perfluorinated compounds—exposure assessment for the general population in Western countries. Int J Hyg Environ Health.

[b14-ehp-118-686] Ghisari M, Bonefeld-Jorgensen EC (2005). Impact of environmental chemicals on the thyroid hormone function in pituitary rat GH3 cells. Mol Cell Endocrinol.

[b15-ehp-118-686] Giesy J, Kannan K (2001). Global distribution of perfluorooctanoate sulfonate in wildlife. Environ Sci Technol.

[b16-ehp-118-686] Gutshall DM, Pilcher GD, Langley AE (1988). Effect of thyroxine supplementation on the response to perfluoro-n-decanoic acid (PFDA) in rats. J Toxicol Environ Health.

[b17-ehp-118-686] Gutshall DM, Pilcher GD, Langley AE (1989). Mechanism of the serum thyroid hormone lowering effect of perfluoro-n-decanoic acid (PFDA) in rats. J Toxicol Environ Health.

[b18-ehp-118-686] Hsieh FY, Bloch DA, Larsen MD (1998). A simple method of sample size calculation for linear and logistic regression. Stat Med.

[b19-ehp-118-686] Hundley S, Sarrif A, Kennedy G (2006). Absorption, distribution and excretion of ammonium perfluorooctanoate (APFO) after oral administration in various species. Drug Chem Toxicol.

[b20-ehp-118-686] Inoue K, Okada F, Ito R, Kato S, Sasaki S, Nakajima S (2004). Perfluorooctane sulfonate (PFOS) and related perfluorinated compounds in human maternal and cord blood samples: assessment of PFOS exposure in a susceptible population during pregnancy. Environ Health Perspect.

[b21-ehp-118-686] Jensen AA, Leffers H (2008). Emerging endocrine disrupters: perfluoroalkylated substances. Int J Androl.

[b22-ehp-118-686] Johnson J, Gibson S, Ober R (1979). Extent and Route of Excretion and Tissue Distribution of Total Carbon-14 in Rats after a Single i.v. Dose of FC-95-14C.

[b23-ehp-118-686] Jones P, Hu W, De coen W, Newsted J, Giesy J (2003). Binding of perfluorinated fatty acids to serum proteins. Environ Toxicol Chem.

[b24-ehp-118-686] Kannan K, Corsolini S, Falandysz J, Fillmann G, Kumar KS, Loganathan BG (2004). Perfluorooctanesulfonate and related fluorochemicals in human blood from several countries. Environ Sci Technol.

[b25-ehp-118-686] Kohrle J, Spanka M, Irmscher K, Hesch R (1988). Flavonoid effects on transport, metabolism and action of thyroid hormones. Prog Clin Bio Res.

[b26-ehp-118-686] Kuklenyik Z, Needham LL, Calafat AM (2005). Measurement of 18 perfluorinated organic acids and amides in human serum using on-line solid-phase extraction. Anal Chem.

[b27-ehp-118-686] Langley AE, Pilcher GD (1985). Thyroid, bradycardic and hypothermic effects of perfluoro-n-decanoic acid in rats. J Toxicol Environ Health.

[b28-ehp-118-686] Lau C, Anitole K, Hodes C, Lai D, Pfahles-Hutchens A, Seed J (2007). Perfluoroalkyl acids: a review of monitoring and toxicological findings. Toxicol Sci.

[b29-ehp-118-686] Lau C, Butenhoff JL, Rogers JM (2004). The developmental toxicity of perfluoroalkyl acids and their derivatives. Toxicol Appl Pharmacol.

[b30-ehp-118-686] Lau C, Thibodeaux J, Hanson R, Rogers J, Grey B, Stanton M (2003). Exposure to perfluorooctane sulfonate during pregnancy in rat and mouse. II: Postnatal evaluation. Toxicol Sci.

[b31-ehp-118-686] Luebker DJ, Case MT, York RG, Moore JA, Hansen KJ, Butenhoff JL (2005). Two-generation reproduction and cross-foster studies of perfluorooctanesulfonate (PFOS) in rats. Toxicology.

[b32-ehp-118-686] Luebker DJ, Hansen KJ, Bass NM, Butenhoff JL, Seacat AM (2002). Interactions of flurochemicals with rat liver fatty acid-binding protein. Toxicology.

[b33-ehp-118-686] Minnesota Department of Health (2008). Health Risk Limits for Perfluorochemicals. Report to the Minnesota Legislature 2008, Minnesota Department of Health Final Report.

[b34-ehp-118-686] Moriyama K, Tagami T, Akamizu T, Usui T, Saijo M, Kanamoto N (2002). Thyroid hormone action is disrupted by bisphenol A as an antagonist. J Clin Endocrinol Metab.

[b35-ehp-118-686] NCHS (National Center for Health Statistics) (2009). National Health and Nutrition Examination Survey: Lab Methods 2005–2006. http://www.cdc.gov/nchs/data/nhanes/nhanes_05_06/pfc_d_met.pdf.

[b36-ehp-118-686] NHANES (2006). Documentation, Codebook, and Frequencies. Surplus Specimen Laboratory Component: Perfluorinated Chemicals (Surplus Sera).

[b37-ehp-118-686] NHANES (2007). Documentation, Codebook, and Frequencies. Laboratory Component: Polyfluorinated Compounds.

[b38-ehp-118-686] Olsen GW, Burlew M, Burris J, Mandel J (2001). A Cross-Sectional Analysis of Serum Perfluorooctanesulfonate (PFOS) and Perfluorooctanoate (PFOA) in Relation to Clinical Chemistry, Thyroid Hormone, Hematology and Urinalysis Results from Male and Female Employee Participants of the 2000 Antwerp and Decatur Fluorochemical Medical Surveillance Program 3M Company. Final Report. Administrative Record AR-226-1087.

[b39-ehp-118-686] Olsen GW, Burris JM, Burlew MM, Mandel JH (2003a). Epidemiologic assessment of worker serum perfluorooctanesulfonate (PFOS) and perfluorooctanoate (PFOA) concentrations and medical surveillance examinations. J Occup Environ Med.

[b40-ehp-118-686] Olsen GW, Burris JM, Ehresman DJ, Froehlich JW, Seacat AM, Butenhoff JL (2007). Half-life of serum elimination of perfluorooctanesulfonate, perfluorohexanesulfonate, and perfluorooctanoate in retired fluorochemical production workers. Environ Health Perspect.

[b41-ehp-118-686] Olsen G, Church T, Miller J, Burris J, Hansen K, Lundberg J (2003b). Perfluorooctanosulfonate (PFOS) and other fluorochemicals in the serum of American Red Cross adult blood donors. Environ Health Perspect.

[b42-ehp-118-686] Olsen GW, Logan PW, Hansen KJ, Simpson CA, Burris JM, Burlew MM (2003c). An occupational exposure assessment of a perfluorooctanesulfonyl fluoride production site: biomonitoring. AIHA J (Fairfax, Va).

[b43-ehp-118-686] Olsen G, Zobel L (2007). Assessment of lipid, hepatic and thyroid parameters with serum perfluorooctanoate (PFOA) concentrations in fluorochemical production workers. Int Arch Occup Environ Health.

[b44-ehp-118-686] Organisation for Economic Co-operation and Development (2005). Results of Survey on Production and Use of PFOS and PFOA, Related Substances and Products/Mixtures Containing These Substances.

[b45-ehp-118-686] Saito N, Harada K, Inoue K, Sasaki K, Yoshinaga T, Koizumi A (2004). Perfluorooctanoate and perfluorooctane sulfonate concentrations in surface water in Japan. J Occup Health.

[b46-ehp-118-686] Santini F, Vitti P, Ceccarini G, Mammoli C, Rosellini V, Pelosini C (2003). *In vitro* assay of thyroid disruptors affecting TSH-stimulated adenylate cyclase activity. J Endocrinol Invest.

[b47-ehp-118-686] Schmutzler C, Gotthardt I, Hofmann PJ, Radovic B, Kovacs G, Stemmler L (2007). Endocrine disruptors and the thyroid gland—a combined *in vitro* and *in vivo* analysis of potential new biomarkers. Environ Health Perspect.

[b48-ehp-118-686] Schröder-van der Elst JP, Smit JW, Romijn HA, van der Heide D (2003). Dietary flavonoids and iodine metabolism. Biofactors.

[b49-ehp-118-686] Scialli A, Iannucci A, Turi J (2007). Combining perfluoroalkane acid exposure levels for risk assessment. Reg Toxicol Pharmacol.

[b50-ehp-118-686] Seacat AM, Thomford PJ, Hansen KJ, Clemen LA, Eldridge SR, Elcombe CR (2003). Sub-chronic dietary toxicity of potassium perfluorooctanesulfonate in rats. Toxicology.

[b51-ehp-118-686] Steenland KJ, MacNeil J, Lally C, Lally A, Vieira V, Fletcher T (2009). Predictors of PFOA levels in a community surrounding a chemical plant. Environ Health Perspect.

[b52-ehp-118-686] Stockholm Convention on Persistent Organic Pollutants (2008). Home page.

[b53-ehp-118-686] U.S. EPA (2006). The 2010/2015 PFOA Stewardship Program.

[b54-ehp-118-686] Vanden Huevel J, Thompson J, Frame S, Gillies P (2006). Differential activation of nuclear receptors by perfluorinated fatty acid analogues and natural fatty acid. Toxicol Sci.

[b55-ehp-118-686] vom Saal FS, Hughes C (2005). An extensive new literature concerning low-dose effects of bisphenol A shows the need for a new risk assessment. Environ Health Perspect.

[b56-ehp-118-686] Weineke N, Neuschafer-Rube F, Bode L, Kuna M, Andres J, Carnevali L (2009). Synergistic acceleration of thyroid hormone degradation by phenobarbitol and the peroxisome proliferation receptor agonist WY14643. Toxicol Appl Pharmacol.

[b57-ehp-118-686] Welshons WV, Thayer KA, Judy BM, Taylor JA, Curran EM, vom Saal FS (2003). Large effects from small exposures. I. Mechanisms for endocrine-disrupting chemicals with estrogenic activity. Environ Health Perspect.

[b58-ehp-118-686] Yu WG, Liu W, Jin YH (2009). Effects of perfluorooctane sulfonate on rat thyroid hormone biosynthesis and metabolism. Environ Toxicol Chem.

